# Investigation of the association between obesity and insulin-induced gene 1 polymorphism at 7q36.3 region in Uygur population in Xinjiang, China

**DOI:** 10.1042/BSR20190498

**Published:** 2019-11-28

**Authors:** Jing Tao, Mayila Abudoukelimu, Xin Shen, Jun Liu, Feng-xia Wang, Jie Yuan, Pei-Pei Gu, Wei Zhu, Xiao-tian Zhang, Zhao Wang, Yi-tong Ma, Guo-qing Li

**Affiliations:** 1 People’s Hospital of Xinjiang Uygur Autonomous Region, Urumqi, Xinjiang, China; 2 Xinjiang Key Laboratory of Cardiovascular Disease Research, Urumqi, Xinjiang, China; 3 Department of Cardiology, the Fifth Affiliated Hospital of Xinjiang Medical University, Urumqi, Xinjiang, China; 4 Department of Cardiology, the First Affiliated Hospital of Xinjiang Medical University, Urumqi, Xinjiang, China

**Keywords:** INSIG1 gene, obesity, polymorphism, susceptibility

## Abstract

**Background:** Obesity is a common heritable trait and a major risk factors of chronic and metabolic diseases. Insulin-induced gene 1 (INSIG1) is known to play important roles in cholesterol and triacylglycerol (TAG) metabolism. In the present study, our primary objective was to explore whether the single nucleotide polymorphisms (SNPs) in INSIG1 gene were associated with obesity in Uygur subjects, in Xinjiang, China.

**Methods:** We designed a case–control study including 516 obese patients and 463 age- and sex-matched control subjects. Three SNPs (rs2721, rs9767875 and rs9719268) were genotyped using TaqMan SNP genotyping assays.

**Results:** For rs2721, the distribution of genotypes, dominant model (GT + TT vs GG), recessive model (TT vs GT + GG) showed significant differences between obese patients and the controls (*P* = 0.008, *P* = 0.005 and *P* = 0.035, respectively). For rs9719268, the distribution of genotypes showed significant differences between obese patients and the controls (*P* = 0.004). The dominant model (GT + TT vs GG) of rs2721 and rs9719268 GT genotype remain significantly associated with obesity after adjustment for confounders (OR = 1.393, 95% CI = 1.047–1.853, *P* = 0.023; OR = 1.631, 95% CI = 1.059–2.512, *P* = 0.026). The TG levels were significantly higher in rs2721 GT/TT genotypes than that in GG genotypes (*P*<0.05).

**Conclusions:** Rs2721 and rs9719268 of INSIG1 gene are associated with obesity in Uygur subjects. Subjects with GT/TT genotype or T allele of rs2721 and GT genotype of rs9719268 were associated with an increased risk of obesity.

## Introduction

Obesity is one of the major risk factors of chronic and metabolic diseases and the prevalence of obesity is increasing worldwide [1–4]. Globally, the proportion of obesity increased from 28.8% in 1980 to 36.9% in 2013 for men and from 29.8% to 38.0% for women in 2013 [5]. Besides, it is estimated that 3.4 million deaths, 3.9% of years of life lost and 3.8% of disability adjusted life years (DALYs) globally were caused by obesity in 2010 [6]. The high prevalence of obesity, together with its severe prognosis reveals it to be an increasing burden.

The etiology and pathogenesis of obesity are likely to comprise a multifactorial disorder, besides the conventional and modifiable risk factors, a large number of studies have demonstrated that obesity is a complicated polygenic disease and the genetic influence accounts for 40–70% of the individual differences [7–10]. In 2007, Frayling et al. performed the first genome-wide association study (GWAS) about BMI, a measure commonly used to define obesity and assess adiposity, and found that the common variants of the fat mass and obesity associated (FTO) gene may affect obesity in the general population and reflect a specific increase in fat mass [11]. Recent GWAS study of body mass index (BMI) have identified 97 genetic variants and all these loci only explained 2.7% of the variance in BMI [12]. While genome-wide estimates suggest that common variation accounts for >20% of BMI variation [12]. Further studies are needed to enrich the genetic knowledge of obesity.

Recently, a multicenter linkage study using a large number of dizygotic twins from six countries reported a linkage susceptibility loci for BMI at 7q36.3 [13]. This region has also been replicated in other studies and been demonstrated to be associated with obesity and triglyceridemia level [14]. The genomic interval on chromosome 7q36.3 includes 22 known genes, including insulin-induced gene 1 (INSIG1), the only biological candidate [15]. INSIG gene, including insig-1 and insig-2, play a lot of important roles in regulating cholesterol or (TAG) synthesis, mainly in the liver [16,17]. However, little is known about the association between insig1 gene and obesity. The present case–control study aimed to explore the association of insig1 gene polymorphisms with obesity in Chinese Uygur subjects.

## Methods

### Subjects

We conducted a case–control study involving Uygur subjects aged 18 years old and above. Obese patients and non-obese controls were all recruited from the Cardiovascular Risk Survey (CRS) [18]. On the basis of BMI, the subjects were classified as normal weight (18.5–22.99 kg/m^2^) and obese (>25 kg/m^2^), according to Asia Pacific population criteria [19]. Individuals with the following conditions were excluded from recruitment: pregnancy; history of cancer, renal or hepatic disease; severe coronary artery disease; substance abuse; diabetes mellitus; corticosteroids or thyroid dosages above replacement dose; and individuals receiving lipid-lowering medications. We finally included 516 obese subjects and 463 normal weight controls in the present study.

Anthropometric measurements (height, weight and waist circumference) were performed using standardized protocols. BMI was calculated as the weight divided by square of height (kg/m^2^). Hypertension was defined as a systolic blood pressure ≥140 mmHg and/or a diastolic blood pressure ≥90 mmHg at least on two distinct occasions. Briefly, diabetes mellitus was defined as two fasting plasma glucose level ≥7.0 mmol/L, the use of insulin or oral hypoglycemic agents, or a self-reported history of diabetes. The following information was collected: age, gender, hypertension, diabetes, total cholesterol (TC), triglyceride (TG), high-density lipoprotein cholesterol (HDL-C) and low-density lipoprotein cholesterol (LDL-C).

### Genotyping

The blood samples were drawn into a 5 ml ethylene diamine tetraacetic acid (EDTA) tube and centrifuged at 4000 × ***g*** for 5 min to separate the plasma content. Genomic DNA was extracted from the peripheral leukocytes using the standard phenol–chloroform method. The DNA samples were stored at -80°C until use. For use, the DNA was diluted to a concentration of 50 ng/μl. Using Haploview 4.2 software and International HapMap Project website phase I and II database (http://www.hapmap.org), we obtained three tag single nucleotide polymorphisms (SNPs) of INSIG1: rs2721, rs9767875 and rs9719268, by using minor allele frequency (MAF) ≥0.05 and linkage disequilibrium patterns with r2≥0.8 as a cutoff. Genotyping was undertaken using the TaqMan® SNP Genotyping Assay (Applied Biosystems). The primers and probes used for the TaqMan® SNP Genotyping Assays (ABI) were chosen based on information on ABI’s website (http://appliedbiosystems.com.cn/). Thermal cycling was conducted using the Applied Biosystems 7900HT Standard Real-Time PCR System. Plates were read using the Sequence Detection Systems (SDS) automation controller software v2.4 (ABI). PCR amplification was performed using 2.5 μl of TaqMan Universa Master Mix, 0.15 μl probes and 1.85 ddH2O in a 6 μl final reaction volume containing 1 μl DNA. Thermal cycling conditions were as follows: 95°C for 5 min; 40 cycles of 95°C for 15 s and 60°C for 1 min. All 96-well plates were read using SDS automation controller software v2.4 (ABI).

### Statistical analysis

The data analysis was performed using SPSS version 17.0 for Windows (SPSS Inc., Chicago, IL, U.S.A.). The Hardy–Weinberg equilibrium (HWE) was assessed via chi-square analysis. The measurement data are shown as the means ± SD, and the differences between the obese subjects and the control subjects were assessed using an independent-sample *t*-test. Differences in the enumeration data, such as the frequencies of smoking, drinking, hypertension between the obese patients and the control subjects were analyzed using the chi-square test. Additionally, logistic regression analyses with effect ratios (odds ratio [OR] and 95% CI) were used to assess the contribution of the major risk factors. Then we examined an additive scale interaction by calculating the relative excess risk due to interaction (RERI), the attributable proportion due to interaction (AP) and the synergy index (SI). A *P* value < 0.05 was considered to be statistically significant

## Result

### Characteristics of subjects

The baseline characteristics of 516 obese patients and 463 control subjects were shown in Table 1. All the measurement data compared by T-test are normally distributed. The mean age, HDL-C, LDL-C levels and the prevalence of male, smoking and drinking were similar between obese patients and the controls (all *P*>0.05). The obese patients and controls differed significantly with regards to BMI(*P* < 0.001), hypertension (*P*<0.001), waist-to-hip ratio (WHR) (*P*<0.001), TC (*P*<0.001) and TG (*P*<0.001).

**Table 1:** Clinical and metabolic characteristics of subjects

Characteristic	Control (*n*=463)	Case (*n*=516)	*P* value
Age (years)	53.37 ± 12.23	53.06 ± 10.64	0.670
Male/female	215/248	233/283	0.688
BMI	21.21 ± 1.23	28.95 ± 3.13	<0.001
Hypertension	115 (24.8)	208 (40.3)	<0.001
Smoking	92 (19.9)	83 (16.1)	0.123
Drinking	42 (9.1)	46 (8.9)	0.932
WHR	0.87 ± 0.07	0.92 ± 0.07	<0.001
TC	4.08 ± 0.97	4.49 ± 1.10	<0.001
TG	1.15 ± 0.65	1.81 ± 1.17	<0.001
LDL-C	2.88 ± 0.89	2.89 ± 0.97	0.768
HDL-C	1.28±0.42	1.25±0.42	0.173

Continuous variables are expressed as mean ± SD. Categorical variables are expressed as percentages.

The *P* value of the continuous variables was calculated by the independent samples *t*-test. The *P* value of the categorical variables was calculated by *χ*^2^ test.

### Association between INSIG1 gene polymorphisms and risk of obesity

Table 2 shows the distribution of genotypes for the three SNPs (rs2721, rs9767875 and rs9719268) of the INSIG1 gene. The genotype distributions of the three SNPs were in accordance with the HWE in all cases and controls (all *P*>0.05). For rs2721, the distribution of the genotypes, the dominant model (GT + TT vs GG), the recessive model (TT vs GT + GG) showed significant differences between obese patients and the controls (*P* = 0.008, *P* = 0.005 and *P* = 0.035, respectively). For rs9719268, the distribution of the genotypes showed significant differences between obese patients and the controls (*P* = 0.004). While, there were no significant differences between obese patients and controls in the distribution of rs9767875 genotypes, dominant model, recessive model and over-dominant model (*P* = 0.974, *P* = 0.833, *P* = 0.877 and *P* = 0.888, respectively).

**Table 2:** Distribution of SNPs of INSIG1 gene in obese patients and non-obese controls

Genotype or allele	Control [*n*(%)]	Case [*n*(%)]	*P* value
rs2721			
GG	307(66.3)	297(57.6)	
GT	138(29.8)	183(35.4)	
TT	18(3.9)	36(7.0)	0.008
*P*^HWE^	0.615	0.287	
Dominant			
GT + TT	156(33.7)	219(42.4)	
GG	307(66.3)	297(57.6)	0.005
Recessive			
TT	18(3.9)	36(7.0)	
GG + GT	445(96.1)	480(93.0)	0.035
Additive			
GT	138(29.8)	183(35.4)	
GG + TT	325(70.2)	333(64.6)	0.060
rs9767875			
GG	285(61.6)	321(62.2)	
GT	150(32.4)	165(32.0)	
TT	28(6.0)	30(5.8)	0.974
*P*^HWE^	0.172	0.158	
Dominant			
GT + TT	178(38.4)	195(37.8)	
GG	285(61.6)	321(62.2)	0.833
Recessive			
TT	28(6.0)	30(5.8)	
GG + GT	435(94.0)	486(94.2)	0.877
Additive			
GT	150(32.4)	165(32.0)	
GG + TT	313(67.6)	351(68.0)	0.888
rs9719268			
GG	421(90.9)	438(84.9)	
GT	42(9.1)	78(15.1)	0.004
*P*^HWE^	0.307	0.063	

The *P* value of the categorical variables was calculated by *χ*^2^ test.

Tables 3 and 4 show the multivariable logistic regression analyses of the major confounding factors for obesity. Following the multivariate adjustments for the confounders, such as age, gender, TG, TC, HDL-C, LDL-C and prevalence of hypertension, smoking and drinking, the dominant model (GT + TT vs GG) of rs2721 remain significantly associated with obesity (OR = 1.393, 95% CI = 1.047–1.853, *P* = 0.023). In addition, carriers of rs9719268 GT genotype had a significantly elevated obesity risk compared with those of GG genotype (OR = 1.631, 95% CI = 1.059–2.512, *P* = 0.026).

**Table 3:** Results of logistic analysis (rs2721)

	OR	95% CI	*P* value
rs2721 (GT + TT vs GG)	1.393	1.047–1.853	0.023
Age	0.982	0.970–0.995	0.007
Gender	0.782	0.565–1.082	0.138
Smoking	0.477	0.290–0.787	0.004
Drinking	1.158	0.628–2.135	0.638
Hypertension	2.138	1.559–2.932	<0.001
TG	2.510	2.013–3.130	<0.001
TC	1.208	1.039–1.404	0.014
HDL-C	0.924	0.653–1.306	0.654
LDL-C	1.079	0.923–1.263	0.339

OR, odds ration; CI, confidence interval.

**Table 4:** Results of logistic analysis (rs9719268)

	OR	95% CI	*P* value
rs9719268 (GT vs GG)	1.631	1.059–2.512	0.026
Age	0.983	0.970–0.995	0.008
Gender	0.773	0.558–1.070	0.121
Smoking	0.49	0.297–0.807	0.005
Drinking	1.09	0.591–2.010	0.782
Hypertension	2.048	1.494–2.807	<0.001
TG	2.54	2.040–3.163	<0.001
TC	1.199	1.032–1.393	0.017
HDL-C	0.925	0.654-1.309	0.66
LDL-C	1.09	0.931-1.276	0.283

OR, odds ration; CI, confidence interval.

### Stratified analysis between INSIG1 gene polymorphisms and obesity risk

We performed stratification analyses in terms of gender, hypertension, smoking, drinking and WHR status to evaluate how these variables modified the association between the SNPs (rs2721, rs9767875 and rs9719268) and obesity risk (Table 5). The rs2721 GT/TT genotypes were shown to significantly increase obesity risk in smokers (AOR = 2.406, 95% CI = 1.194–4.850, *P* = 0.014) and drinkers (AOR = 2.710, 95% CI = 1.575–5.710, *P* = 0.008). A significant interaction between rs2721 and smoking (RERI = 0.61, AP = 0.40 and SI = 5.1, *P*<0.05), drinking (RERI = 1.4, AP = 0.56 and SI = 12.67, *P*<0.05) were also observed. The rs9719268 GT genotype were shown to significantly increase obesity risk in females (AOR = 2.234, 95% CI = 1.240–4.025, *P* = 0.007), nonsmokers (AOR = 2.085, 95% CI = 1.276–3.408, *P* = 0.003), nondrinkers (AOR = 1.806, 95% CI = 1.137–2.870, *P* = 0.012) and high WHR (AOR = 1.747, 95% CI = 1.030–2.961, *P* = 0.038).

**Table 5:** Stratifed analysis between INSIG1 gene polymorphisms and obesity risk

Variables	rs2721 (control/case)		Adjusted Ora	*P*^1^	RERI	AP	SI	rs9767875 (control/case)		Adjusted Ora	*P*^1^	RERI	AP	SI	rs9719268 (control/case)		Adjusted Ora	*P*^1^	RERI	AP	SI
	GG	GT + TT	95% CI					GG	GT + TT	95% CI					GG	GT					
**Gender**					0.04	0.026	0.93					0.36	0.34	0.16					0.91	0.40	3.68
**Females**	165/171	83/112	1.399(0.949–2.062)	0.090				153/165	95/118	1.148(0.784–1.680)	0.479				228/236	20/47	2.234(1.240–4.025)	0.007			
**Males**	142/136	73/97	1.327(0.868–2.027)	0.191				132/156	83/77	0.784(0.511–1.204)	0.267				193/202	22/31	1.085(0.564–2.087)	0.808			
**Hypertension**					1.23	0.34	1.89					0.17	0.08	1.2					1.47	0.37	1.97
**No**	221/176	127/132	1.274(0.907–1.790)	0.162				216/198	132/110	0.968(0.686–1.366)	0.854				317/267	31/41	1.471(0.859–2.522)	0.16			
**Yes**	86/131	29/77	1.536(0.881–2.676)	0.130				69/123	46/85	0.926(0.551–1.555)	0.771				104/171	11/37	1.916(0.890–4.127)	0.097			
**Smoking status**					0.61	0.40	5.1*					0.25	0.29	0.36					1.83	4.16	0.79
**Never**	240/262	131/171	1.206(0.881–1.651)	0.243				224/270	147/163	0.932(0.683–1.272)	0.655				342/361	29/72	2.085(1.276–3.408)	0.003			
**Ever**	67/45	25/38	2.348(1.163–4.742)	0.017				61/51	31/32	1.232(0.613–2.477)	0.558				79/77	13/6	0.584(0.197–1.731)	0.332			
**Drinking status**					1.40	0.56	12.67*					0.22	0.2	0.57					1.47	2.01	0.23
**Never**	273/281	148/189	1.222(0.908–1.643)	0.186				256/291	165/179	0.941(0.701–1.264)	0.688				387/398	34/72	1.806(1.137–2.870)	0.012			
**Ever**	34/26	8/20	2.710(1.575–5.710)	0.008				29/30	13/16	1.738(0.554–5.456)	0.344				34/40	8/6	0.973(0.225–4.211)	0.971			
**WHR**					1.22	0.18	1.26					0.15	-0.04	0.95					3.56	0.42	1.91
**Normal**	125/36	61/30	1.512(0.798–2.865)	0.205				121/46	65/20	0.781(0.395–1.541)	0.476				168/57	18/9	1.518(0.565–4.076)	0.408			
**High**	182/271	95/179	1.256(0.892–1.769)	0.192				164/275	113/175	0.911(0.652–1.274)	0.587				253/381	24/69	1.747(1.030–2.961)	0.038			

OR, odds ration; CI, confidence interval.

1 Adjusted for the confounders, such as age, gender, TG, TC, HDL-C, LDL-C and prevalence of hypertension, smoking and drinking.

* *P*<0.05.

### Associations between haplotypes of INSIG1 gene polymorphisms and obesity risk

In the present study, we investigated the interaction of INSIG1 polymorphisms in obesity through linkage disequilibrium analysis and the results showed the strong linkage disequilibrium among INSIG1 rs2721, rs9767875 and rs9719268 polymorphisms. The associations between possible haplotypes and risk of obesity were estimated and listed in Table 6. The INSIG1 rs2721/ rs9767875/ rs9719268 haplotype G-G-T and T-G-G were significantly associated with increased risk of obesity (OR = 1.984, 95% CI = 1.186-3.320, *P* = 0.008; OR = 1.512, 95% CI = 1.170–1.953, *P* = 0.001). However, the haplotype G-G-G was associated with lower risk of obesity (OR = 0.751, 95% CI = 0.624–0.903, *P* = 0.002).

**Table 6:** Associations between haplotypes of INSIG1 gene polymorphisms and obesity risk

Haplotype	Case	Control	OR(95% CI)	*P*
GGG	571.37(0.554)	579.09(0.625)	0.751(0.624–0.903)	0.002
GGT	47.06(0.046)	21.98(0.024)	1.984(1.186–3.320)	0.008
GTG	150.27(0.146)	137.78(0.149)	0.985(0.766–1.266)	0.904
TGG	176.20(0.171)	112.16(0.121)	1.512(1.170–1.953)	0.001
TTG	56.17(0.054)	54.96(0.059)	0.920(0.627–1.350)	0.671

Haplotype frequency < 0.03 in both obese patients and controls has been dropped; OR, odds ratio; 95% CI, 95% confidence interval.

### Genotypes and serum lipid levels

**Figure 1:**
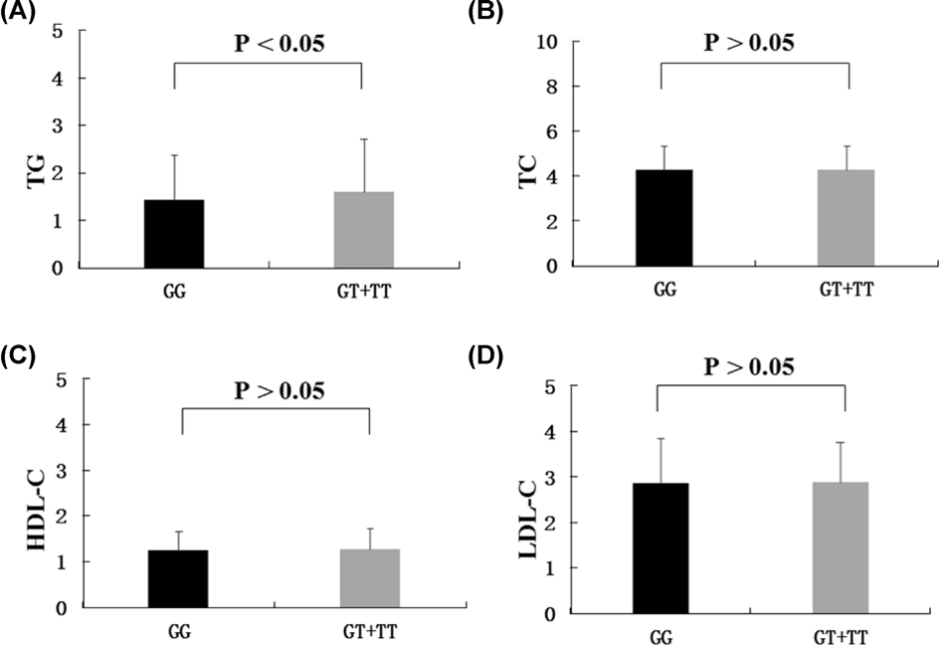
Association between rs2721 and lipid parameters (**A**) The TG levels were significantly higher in rs2721 GT/TT genotypes than that in GG genotypes (*P*<0.05). (**B**) There exists no significant difference of TC between rs2721 GT/TT genotypes and GG genotypes (*P*>0.05). (**C**) There exists no significant difference of HDL-C between rs2721 GT/TT genotypes and GG genotypes (*P*>0.05). (**D**) There exists no significant difference of LDL-C between rs2721 GT/TT genotypes and GG genotypes (*P*>0.05).

As shown in Figure 1, the TG levels were significantly higher in rs2721 GT/TT genotypes than that in GG genotypes (*P*<0.05).

## Discussion

In the present study, we investigated associations between three SNPs in the INSIG1 gene and obesity risk in a Chinese Uygur population. Our results show that rs2721 and rs9719268 are significantly associated with obesity susceptibility.

Obesity is a common heritable trait and a major risk factor for the development of hypertension, diabetes, coronary heart disease and stroke [20–25]. Long-term trends of overweight indicate a strong impact of lifestyle on the risk of obesity, despite this, many studies that aim to evaluate family heredity have proven the influence of genetic factors on BMI [13,26]. Recently, Kettunen performed a large multicenter linkage study including 4401 twin families from six countries (Australia, Denmark, Holland, Finland, Sweden and the United Kingdom) on BMI and found suggestive evidence for a quantitative trait locus on 7q36.3 [13]. It is very interesting that a recent genome-wide linkage analysis on BMI in 126 Chinese dizygotic twins identified a genome-wide significant linkage peak on 7q36.3 too [26]. According to bioinformatics analysis, we found that there exists genes directly related to glycolipid metabolism in the 7q36 region, such as INSIG1, the only biological candidate.

Insulin-induced genes are newly characterized genes in human and encode two isoform membrane proteins in the endoplasmic reticulum (ER): INSIG1 and INSIG2. Previous studies have shown that INSIG1 genes can affect intracellular cholesterol synthesis through sterol regulatory element binding proteins (SREBP) pathway and sterol-regulated degradation of HMG-CoA reductase (HMGCR) pathway, two of the most important mechanisms governing the negative feedback regulation of *de novo* cholesterol biosynthesis. SREBP is a transcription factor located in the ER and its main function is to control the synthesis of cholesterol and fatty acids [27,28]. When the cellular sterol levels rise, INSIG1 binds to the sterol-sensing domain (SSD) of sterol regulatory element-binding protein (SREBP) cleavage-activating protein (SCAP), blocking the binding of SCAP to SREBP and preventing the transport of the SCAP/SREBP complex from ER to the Golgi, causing transcriptional rates of SREBP target genes to decline and ultimately leading to a reduction in cholesterol synthesis and uptake. When cells are depleted of sterols, INSIG1 expressed at a low level, the binding of INSIG1 and SCAP is reduced and SREBPs are transported by SCAP from ER to Golgi, where they are processed and released into the cytosol on their way to the nucleus to activate transcription of lipogenic genes, and finally leading to the increasing of cholesterol synthesis [29,30]. HMGCR, the enzyme that catalyzes the rate-determining step of the cholesterol biosynthetic pathway, is the rate-limiting enzyme in the biosynthesis of cholesterol. Binding of HMGCR to insig proteins leads to the ubiquitination and degradation of the reductase. The degradation slows down the reductase-catalyzed conversion of HMG-CoA to mevalonate, a rate-limiting step in cholesterol synthesis [31–32]. Through these two mechanisms, INSIG1 protein cause coordinated association in both transcription of relevant genes and sterol pathway activity.

Studies conducted in animals have demonstrated the critical roles of INSIG1 gene in lipid homeostasis. *In vivo*, the mRNA level of INSIG1 gene in fat tissue of diet-induced obese rats increased significantly [32]. Takaishi et al. infected Zucker diabetic fatty (ZDF) (fa/fa) rats with recombinant adenovirus containing insig-1 cDNA to overexpress the INSIG1 gene and found that the increase in endogenous insig-1 expression was associated with augmented lipogenesis. Overexpression of INSIG1 gene was shown to reduce high levels of TG in both liver and plasma of Zucker diabetic fatty rats [33]. Moreover, single knockout of either INSIG1 or INSIG2 gene alone in mouse may result in the increasing of cholesterol and TG level in mouse livers [34]. All these suggested that INSIG1 gene may affect the homeostasis of lipid.

The association between INSIG1 gene polymorphisms and obesity was poor. Liu et al. performed a case–control study including 705 obese cases and 1325 non-obese controls to explore the association of genetic variants in INSIG-SCAP-SREBP pathway with obesity in Chinese children [35]. And the result revealed that rs2721 and rs13223383 have no association with obesity [35]. However, Le Hellard et al. found that INSIG1 rs13223383 was related to BMI change in Germans [36]. In addition, Smith analyzed a quantitative trait locus (QTL) for plasma TG levels [logarithm of odds (LOD) = 3.7] on human chromosome 7q36.3 and examined 29 SNPs across INSIG1 gene [15]. They found that the INSIG1 rs2721 was associated with TG levels. Compared with GG genotype, carriers of INSIG1 rs2721 TT genotype had 9% higher TG levels and 2-fold lower expression of INSIG1 in surgical liver biopsy samples [15].

In the present study, we genotyped polymorphisms of rs2721, rs9767875 and rs9719268 in INSIG1 gene and found that rs2721 and rs9719268 were associated with obesity. The rs2721 GT/TT genotype has a higher frequency in obese patients than that in controls. After adjustments for several confounders, this association remained exist, indicating that the rs2721 GT/TT were independent risk factors for obesity and the risk of obesity was increased in the subjects with the T allele in rs2721. The rs9719268 GT genotype has a higher frequency in obese patients than that in controls. After adjustments for confounders, this association remained exist, indicating that the rs9719268 GT were independent risk factors for obesity and the risk of obesity was increased in the subjects with the T allele in rs9719268.

Furthermore, we also analyzed the relationship between genotypes and lipid levels. We found that T allele (GT/TT) carriers in rs2721 have higher levels of TG when compared with T allele non-carriers. This finding is consistent with our previous conclusions. We drew the inference that variation at rs2721 in INSIG1 gene may influence lipid homeostasis and lead to the increased total content of TG. Th e mechanism may be that INSIG1 genes can affect intracellular cholesterol synthesis through SREBP pathway and sterol-regulated degradation of HMGCR pathway, two of the most important mechanisms governing the negative feedback regulation of *de novo* cholesterol biosynthesis. Despite the promising findings in the present study, several limitations should be mentioned. First of all, we only draw conclusions based on the present observational association study, we failed to get a cause-and-effect relationship between risk factors and obesity. Secondly, the sample size of present study is relatively moderate. The association between rs2721 and rs9719268 and obesity should be confirmed by studies with larger sample size. Thirdly, the present study lacked functional validation. Additional studies need to be undertaken to clarify the underlying molecular mechanism between INSIG1 gene polymorphisms and obesity.

## Conclusion

This study revealed that rs2721 and rs9719268 of INSIG1 gene are associated with obesity in Uygur subjects. Subjects with GT/TT genotype or T allele of rs2721 and GT genotype of rs9719268 were associated with an increased risk of obesity.

## Availability to Data and Materials

All data generated or analyzed during this study are included in this published article.

## Abbreviations

BMI: body mass index
ER: endoplasmic reticulum
FTO: fat mass and obesity associated
GWAS: genome-wide association study
HDL-C: high-density lipoprotein cholesterol
HMGCR: HMG-CoA reductase
HWE: Hardy–Weinberg equilibrium
INSIG: insulin-induced gene
LDL-C: low-density lipoprotein cholesterol
SCAP: SREBP cleavage-activating protein
SNPs: single nucleotide polymorphisms
SREBP: sterol regulatory element binding proteins
TAG: triacylglycerol
TC: total cholesterol
TG: triglyceride
WHR: waist-to-hip ratio
